# Pratique anesthésique à Lubumbashi: indications, types de chirurgie et types de patient

**DOI:** 10.11604/pamj.2015.21.240.6091

**Published:** 2015-08-04

**Authors:** Alain Kabey a Kabey, Muyumba Lubanga, Mundongo Tshamba, Mukeng Kaut, Kaij Kakambal, Manika Muteya, Kilembe Manzanza, Kapend Kalal

**Affiliations:** 1 Faculté de Médecine, Service d'Anesthésie et Réanimation, Cliniques Universitaires de Lubumbashi, Université de Lubumbashi, Lubumbashi, République Démocratique du Congo; 2 Hôpital Central de Ndola, Republique de Zambie; 3 Faculté de Médecine, Département de Santé Publique, Université de Lubumbashi, République Démocratique du Congo; 4 Faculté de Médecine, Département d'Anesthésie Réanimation, Université de Kinshasa, République Démocratique du Congo; 5 Faculté de Médecine, Département de Médecine Interne, Cliniques Universitaires de Lubumbashi, République Démocratique du Congo

**Keywords:** Pratique anesthésique, types de chirurgie, patients, Lubumbashi, anesthetic practice, types of surgery, patients, Lubumbashi

## Abstract

**Introduction:**

Cette étude a pour objectif de décrire la pratique anesthésique dans un pays à faible revenu et où le plateau technique anesthésique est moins équipé.

**Méthodes:**

Une étude descriptive transversale a été menée durant l'année 2013. L'enquête a concerné les pratiques anesthésiques, les indications chirurgicales et les caractéristiques des malades. L'encodage et l'analyse des données ont été réalisées grâce aux logiciels Epi Info 3.5.3 et Excel 2010.

**Résultats:**

Nous avons enregistré 2358 patients dont l’âge médian était de 29 + 15 ans, avec 81,5% âgés de 11 à 50 ans. Parmi eux, 67,3% des malades étaient du sexe féminin. Dans ensemble, 62,5% de ces patients étaient pris en charge pour les interventions programmées. L’évaluation du risque anesthésique a montré que 91,9% des patients étaient de la classe ASA I et II. La chirurgie la plus pratiquée était viscérale (46,7%) suivie de la chirurgie gynéco-obstétricale (29,2%). Les différents types d'anesthésie étaient les suivants: anesthésie générale (87,6%), locorégionale (11,8%) et combinée (0,6%).

**Conclusion:**

La pratique anesthésique dans la population d’étude était dominée par l'anesthésie générale. Les malades étaient au trois quart de sexe féminin et de la classe ASA I et II. Les résultats de cette étude indiquent la nécessité d’évaluer l'issue de cette pratique. La pratique anesthésique à Lubumbashi est tributaire du plateau technique, des compétences du personnel et de l'acceptabilité du type d'anesthésie par les patients.

## Introduction

Dans certains pays d'Afrique, la pratique de l'anesthésie est méconnue et n'est pas évaluée [1]; alors que l'anesthésie de par sa place en cours d'intervention chirurgicale est un soutien important à l’équipe chirurgicale, comme le montre l’étude de Chu à l'Est de la république démocratique du Congo [2]. Elle permet un bon déroulement de l'intervention avec une suppression de la douleur, une immobilité du malade ainsi qu'une protection neurovégétative [3]. La place de cette pratique auprès des chirurgiens n'est pas négligeable. Il y a lieu d’évaluer ses indications et son apport durant l'acte opératoire. La France, l'Italie et l'Espagne ont eu à faire une évaluation pareille qui a permis de voir l’évolution sur le temps et d'améliorer le fonctionnement des services d'anesthésie et réanimation [3–7]. Le contexte particulier de l'Afrique dominé par une pénurie de médecins anesthésistes et une capacité insuffisante de formation en cours d'emploi justifient la dégradation de la prise en charge anesthésique et augurent d'un avenir sombre [6–11]. A Lubumbashi, sur les différents sites où nous avons mené l’étude, il y a 15 IADE (infirmiers anesthésistes diplômés d'Etat, 4 internes en anesthésie et réanimation et un seul anesthésiologiste. Dans l'ensemble cinq salles d'opération existent dans un site d’étude, trois sur deux sites et deux sur un site, tandis que pour le reste il n'y a qu'une salle d'opération par site. Une autre piste de solution qui est utilisée comme alternative, est le recours à l'anesthésie locorégionale, étant donné qu'elle est moins coûteuse et plus accessible dans cet environnement [12–14]. Dans le cas particulier de la République Démocratique du Congo et concernant la situation dans l'Est du pays, cette pénurie en personnel qualifié se traduit par une incapacité de répondre convenablement aux besoins chirurgicaux et anesthésiques qui sont brusquement accrus des suites des conflits armés [4]. Ainsi, cette étude a pour objectif de décrire la pratique anesthésique (indications, types de chirurgie et profil des patients pris en charge) dans cet environnement précaire caractérisé par un plateau technique très limité, une insuffisance de personnel qualifié compétent et une demande accrue de soins de santé en général et de l'anesthésie en particulier.

## Méthodes

Cette étude est une enquête descriptive transversale dans la ville de Lubumbashi durant l'année 2013. Elle a concerné les sept communes et porte sur 36 centres dont les données sont traitées strictement de façon anonyme. Etait inclus tout malade ayant subi une intervention chirurgicale sous la supervision d'un personnel attaché exclusivement à l'administration de l'anesthésie dans une salle d'opération. La collecte des données était faite par le préposé de l'anesthésie au cours de la consultation pré anesthésique et en cours d'anesthésie à l'aide d'un questionnaire représenté par une fiche préétablie adressée aux différents prestataires dans les centres susmentionnés. Les paramètres suivants étaient concernés: l’âge, le sexe, la classification de l'American Society of Anesthesiologist (ASA), le type de chirurgie, le type d'anesthésie et ses variantes. La version finale de cette fiche était le résultat d'une harmonisation à la suite d'un pré-test. La réalisation de cette analyse a nécessité un référentiel standardisé que les enquêteurs ont utilisé durant cette investigation. L'encodage et la saisie ont été faits à l'aide du logiciel Epi info 2005 version 3.5.1, le traitement et l'analyse grâce aux logiciels SPSS 11.0 et Excel 2007.

## Résultats

### Profil des patients

**Figure 1:**
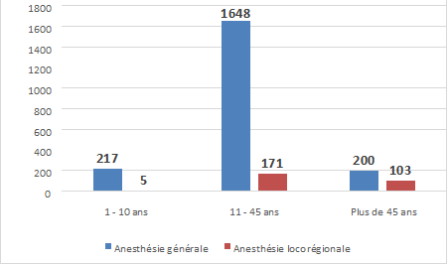
Type d'anesthésie versus l’âge des patients

**Figure 2:**
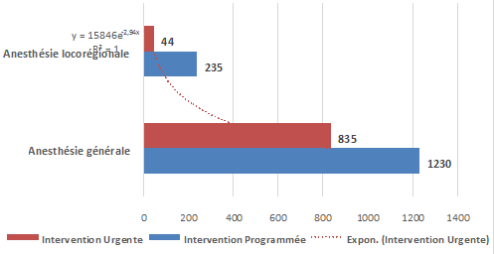
Type d'anesthésie versus type d'intervention

**Figure 3:**
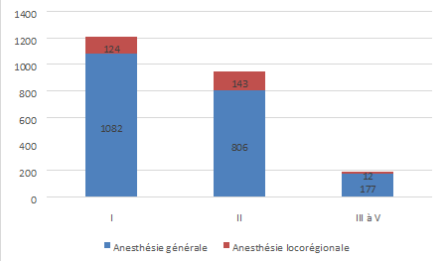
Type d'anesthésie versus classification ASA

Sur les 37 centres identifiés où se pratique l'anesthésie,36 (97,3%) ont accepté la réalisation de cette étude. Au total, 2358 patients ont été recensés et leurs dossiers analysés. Le tableau 1 indique que deux patients sur trois (67,3%) étaient du sexe féminin qui avait par ailleurs été beaucoup plus soumis à l'anesthésie générale (Χ^2^ =190,02; p = 0,000). La Figure 1 montre que 9,4% des patients étaient âgés de moins de 11 ans contre 81,5% qui étaient dans la tranche d’âge de 11 à 50 ans et le reste des malades étaient âgés de plus de 51 ans et avaient plus significativement subi l'anesthésie générale que l'anesthésie locorégionale(Χ^2^ = 171,59; p = 0,000). Les interventions programmées et les urgences chirurgicales avaient respectivement représenté 62,5% et 27,5% des cas (Figure 2). Quant à la classification ASA, 91,9% des patients étaient classés ASA I et II versus 8,1% d'entre eux relevaient des classes ASA III à V (Figure 3).

**Tableau 1:** Types d'anesthésie versus le sexe des opérés

Sexe des Patients	Types d'anesthésie		Total
	Anesthésie générale	Anesthésie locorégionale	
Féminin	1489 (72,1%)	86 (30,8%)	1575 (67,2%)
Masculin	576 (27,9%)	193 (69,2%)	769 (32,8%)
TOTAL	2065	279	2344 (100%)

### Types de chirurgie

**Figure 4:**
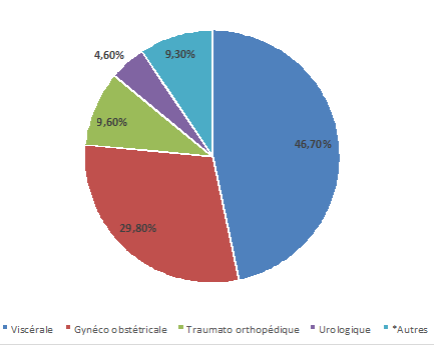
Types de chirurgie réalisée

Les résultats de la Figure 4 indiquent que 46,7% des interventions concernaient la chirurgie digestive, 29,8% la chirurgie gynéco-obstétricale, 9,6% la chirurgie traumato-orthopédique, 4,6% la chirurgie urologique et 9,3% les autres types (plastique, ORL, ophtalmologique, maxillo-faciale et stomatologique, neurochirurgical et vasculaire). La chirurgie majeure était le type de chirurgie le plus pratiqué (68,9% des cas) à Lubumbashi, avec ouverture des grandes cavités du corps et altérations des fonctions vitales.

### Types d'anesthésie

**Figure 5:**
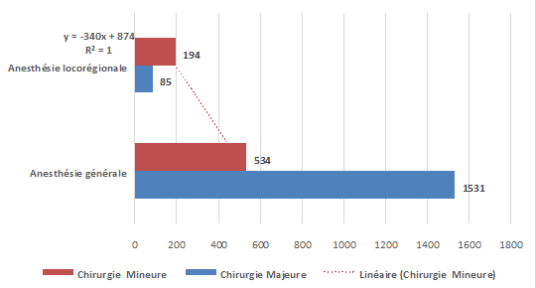
Type d'anesthésie versus type de chirurgie

L'analyse de types d'anesthésie pratiquée indique que l'anesthésie générale était réalisée dans 86,7% des cas contre 11,8% pour l'anesthésie locorégionale et 0,6% pour l'anesthésie combinée. La majorité de l'anesthésie locorégionale est constituée de la rachianesthésie (66%). Les résultats du Tableau 1 indiquent que les femmes étaient fréquemment opérées sous anesthésie générale (72,1%), alors que les malades de sexe masculin qui l’étaient sous anesthésie locorégionale (69,2%). Les deux types d'anesthésie générale et locorégionale étaient un peu plus pratiqués sur les patients âgés de 11 à 45 ans que sur les patients d'autres groupes d’âge (Figure 1). Cependant si l'on considère le taux d'utilisation, les patients de plus de 45 ans ont présenté un taux d'anesthésie locorégionale de loin supérieur à ceux des deux autres groupes d’âge (34% versus 2.3% pour 1-10 ans et 9.4% pour 11-45 ans). Les trois quart des patients opérés dans cet échantillon pour les interventions non urgentes subissent l'anesthésie locorégionale, tandis que les urgences sont prises en charge sous anesthésie générale dans 95% des cas (Figure 2). La majorité des patients quelque soit leur classe ASA sont opérés sous anesthésie générale et pour ceux de la classe ASA I et II, ils sont pris sous anesthésie locorégionale, dans la proportion respective de 10% et 15%. Les patients ayant des pathologies altérant l’état général, faisant partie de la classe ASA III à V sont rares (8,1%) et sont souvent pris sous anesthésie générale (Figure 3). Les chirurgies majeures sont faites dans la grande proportion des cas sous anesthésie générale (Figure 5).

## Discussion

L'analyse des pratiques anesthésiques est quasi inexistante dans la ville de Lubumbashi. La réalisation de cette étude permet d’établir un état de lieu et de constituer une base des données. Les praticiens de l'anesthésie sont dans la plupart des cas un personnel qui n'a pas suivi une formation académique structurée, généralement des médecins et infirmiers dévoués à réaliser l'anesthésie en milieu hospitalier. Etant donné que les principes de base de la pratique de l'anesthésie restent les mêmes et qu'il existe des adaptations propres à chaque milieu [6, 11–13], cette étude s'inscrit dans la logique d'analyser ces adaptations dans un pays à faible revenu. En ce qui concerne le profil des patients à Lubumbashi, il ressort des résultats de cette étude que 67,8% sont de sexe féminin et 32,2% de sexe masculin. Ces résultats sont relativement différents par rapport à ceux observés dans l’étude française d'Auroy qui avait trouvé que 55% des patients étaient de sexe féminin versus 45% de sexe masculin [7]. Dans une étude italienne, Peduto et collaborateurs avaient observé 54% des patients de sexe féminin contre 46% pour le sexe masculin [5]. Pour Sabaté et collaborateurs en Espagne, 58% des malades étaient de sexe féminin [6]. Ces observations seraient liées aux types des pathologies, à l'indication chirurgicale et le plateau technique mis à la disposition du personnel anesthésiste dans les structures des soins de santé. Toutes proportions gardées, toutes les autres études confirment une proportion plus élevée pour les patients de sexe féminin que pour leurs homologues de sexe masculin; ces observations seraient très probablement liées à la fréquence élevée des interventions de gynéco-obstétrique. En ce qui concerne le type d'anesthésie pratiquée, nos résultats indiquent une prédominance de l'anesthésie générale. Cette observation s'explique de prime abord par le choix et la maîtrise de cette technique par les prestataires dans la ville de Lubumbashi. Par ailleurs, les malades de sexe féminin ont une préférence pour l'anesthésie générale contrairement à ceux du sexe masculin qui se prêtent volontiers à l'anesthésie locorégionale. Si l'indication chirurgicale n'est pas une orientation absolue, cette participation active du patient est prépondérante sur le choix du type d'anesthésie à pratiquer. La tranche d’âge de 11 à 45 ans est la plus concernée par l'anesthésie générale parce que c'est la pratique standard la mieux maîtrisée par tous les praticiens de l'anesthésie pour cette catégorie des patients. L'anesthésie locorégionale est rarement pratiquée chez les malades de moins de 45 ans et plus pratiquée chez les plus de 45 ans que l'anesthésie générale à cause de leur fragilité relative, à l'acceptabilité et si des comorbidités sont observées. Nos observations corroborent les études antérieures à ce sujet [11–13]. La répartition des malades par tranche d’âge indique que l’âge moyen est de 29 ± 15 ans avec une prédominance des patients âgés de 11 à 45 ans. En France et en Italie, les résultats de recherche indiquent des proportions divergentes avec 33% des malades âgés de plus de 65 ans en France versus 28,4% pour la même tranche d’âge en Italie [5, 7]. Ces observations européennes seraient liées au fait que ces études n'ont pas utilisé les mêmes distributions d’âge. Les observations faites à Lubumbashi seraient liées à l'espérance de vie qui est inférieure à 45 ans, le manque de technicité pour la prise en charge de ces malades qui présentent beaucoup des comorbidités et aussi le refus de certaines personnes âgées d’être opérées. En plus, les individus de cette tranche d’âge de plus de 25 ans sont actifs et possèdent les moyens financiers qui facilitent l'accès aux soins, dans ce pays à faible revenu, où aucun système d'assurance maladie ou mutuelle des soins de santé n'existe.

Les patients de la ville de Lubumbashi sont anesthésiés dans la majorité des cas pour les interventions chirurgicales programmées et mineures, qui sont faites sous anesthésie locorégionale. Tandis que les cas urgents qui sont des chirurgies majeures, les sont sous anesthésie générale. Le type des malades anesthésiés sont plus des classes ASA I et II dans la proportion de 91,4%, contre 8,6% pour les classes ASA III à V. En France, une étude indique 88% des patients de la classe I et II. En Italie, ils représentent 79% des cas contre 21%pour les classes ASA III à V [5, 7]. Cette observation indique qu’à Lubumbashi les patients pris en charge sont plus en bon état général qu'en France et en Italie. Dans ces pays, la France et l'Italie, le vieillissement et l'allongement de l'espérance de vie de la population prédisposent les personnes âgées de plus en plus à des défaillances organiques dues à l’âge. Par ailleurs, cet état de fait peut profiter de la disponibilité d'un équipement et des compétences appropriés pour prendre en charge des cas de plus en plus graves et les chirurgies majeures dans ces deux pays. Ce contexte de travail n'est pas transposable dans la ville de Lubumbashi. Les types de chirurgie représentés par notre population d’étude sont par ordre de fréquence: la chirurgie viscérale (47,9%) qui est la conséquence des syndromes infectieux, mais aussi, suite à beaucoup d'appendicectomie, suivi de la chirurgie obstétricale dans 20,1% des cas. La chirurgie gynécologique (9,4%) s'explique par le fait que la population est jeune et pro nataliste (âge modale 28 ans) et sexuellement active. Cette observation s'explique aussi par la chirurgie gynécologique avec les cas de grossesse extra-utérine et abcès tubo-ovarien. La chirurgie orthopédique et traumatologique (9,3%) est tributaire du développement industriel de la ville, des besoins en soins de santé des ouvriers qui subissent des lésions sur le lieu de travail, mais aussi par les accidents du trafic routier. Tous les autres types de chirurgie représentent 23,0%, soit moins du tiers de tous les cas, seraient liés au manque d’équipement et de compétences, ce qui oblige le transfert des patients vers les centres les mieux équipés. En France, c'est la chirurgie obstétricale qui arrive en tête (33%) suivie de la viscérale avec 18,5% des cas [7]. En Italie, la chirurgie viscérale concerne18,5% des cas tandis que la chirurgie orthopédique et traumatologique est pratiquée chez 16,7% des patients [5]. En Espagne, les chirurgies orthopédique, ophtalmologique et viscérale représentent respectivement 18, 15, 9 et 14,9% des cas [6]. Le type de chirurgie qui est fréquemment réalisé à Lubumbashi est la chirurgie majeure. Elle est généralement réalisée sous anesthésie générale dans 74,1%, tandis que la mineure l'est sous anesthésie locorégionale dans 69,5% des cas. Cependant, en France et en Espagne ces proportions deviennent 65% pour l'anesthésie générale et 32,8% pour l'anesthésie locorégionale; pour la chirurgie majeures [6, 7]. La maîtrise des techniques d'anesthésie locorégionale et la disponibilité des kits de travail permettent en France et en Espagne la pratique de l'anesthésie locorégionale contrairement à Lubumbashi, où ce type de pratique d'anesthésie locorégionale est assez rare. En effet dans cette ville, à l'instar d'autres régions d'Afrique [13–16], la difficulté d'accès aux drogues et aux aiguilles spécifiques utilisables pour l'anesthésie locorégionale ainsi que le manque de stimulateur électrique, équipement qui permet d'améliorer l'efficacité de l'anesthésie locorégionale dans la plupart des structures, expliquent la limitation de la pratique de l'anesthésie locorégionale.

## Conclusion

Cette étude donne des informations pertinentes sur les caractéristiques des malades, les indications et les types de chirurgie ainsi que les types d'anesthésies pratiquées. La pratique anesthésique à Lubumbashi est tributaire du plateau technique, des compétences du personnel et de l'acceptabilité du type d'anesthésie par les patients. Ces résultats indiquent la nécessité d’évaluer la pratique anesthésique et la mise en route des reformes pour l'amélioration de la pratique anesthésique à Lubumbashi.

## References

[1] Lokossou T, Zoumenou E, Secka G, Ouro Bang'na F, Le Polain de Waroux B, Veyckemans F, Baele P, Chobli M (2007). Anesthesia in French-speaking sub-Saharan Africa: an overview. Acta Anæsthesiologica belgica..

[2] Chu K, Havet P, Ford N, Trelles M (2010). Surgical care for the direct and indirect victims of violence in the eastern Democratic Republic of Congo. Confl Health..

[3] Pinsker M (1986). Anesthésia: a pragmatic construct. Anesth Analg..

[4] Clergue F, Auroy Y, Pequignot F, Jougla E, Lienhart A, Laxenaire LC (1999). French survey of anesthesia in 1996. Anesthesiology..

[5] Peduto V, Chevallier P, Casati A, Chevallier (2004). A multicenter survey on anaesthesia practice in Italy. Minerva Anestesiol..

[6] Sabate S, Canet J, Gomar C, Castillo J, Villalonga A (2008). Etude transversale de la pratique de l'anesthésie en Catalogne, Espagne. Ann Fr Anesth Reanim..

[7] Auroy Y, Laxenaire M, Clergue F, Péquinot F, Jougla F, Lienhart A (1998). Anesthésie selon les caractéristiques des patients, des établissements et de la procédure associée. Annales françaises d'anesthésie et de Réanimation..

[8] Sanou I, Vilasco B, Obey A, Binam F, Chobli M, Tourc MK, Adnet P (1999). Evolution de la démographie des praticiens d'anesthésie en Afrique francophone au sud du Sahara. Annales françaises d'anesthésie et de réanimation..

[9] Van Houwe P (2007). Anesthesia in Africa: quo vadis?. Acta Anaesthesiol Belg..

[10] Adnet P, Diallo A, Sanou J, Chobli M, Murat I, Fian E (1999). Pratique de l'anesthésie par les infirmier(e)s en Afrique francophone subsaharienne. Annales françaises d'anesthésie et de réanimation..

[11] Ouro-Bang'na Maman A (2003). Anesthetic practice in developing country: the view from Lomé in Togo. World Anesthesia..

[12] Okafor UV, Ezegwui HU, Ekwazi K (2009). Trends of different forms of anaesthesia for caesarean section in South-eastern Nigeria. Journal of Obstetrics and Gynecology..

[13] Nganga JL, Bikandou G, Massengo R, Mbemba M, Guo X (1997). Le choix d'une anesthésie pratique adaptée à notre environnement chirurgical. Médecine d'afrique noire..

[14] Binam F, Lemardeley P, Blatt A, Arvis T (1999). Pratiques anesthésiques à Yaoundé (Cameroun). Annales Françaises d'anesthésie et de réanimation..

[15] Bashford T (2014). Anaesthesia in Ethiopia: providers’ perspectives on the current state of the service. Tropical Doctor..

[16] Keïta M, Doumbia D, Goïta D, Coulibaly Y (2013). Pratique de l'anesthésie locorégionale à propos de 1261 cas. Mali Médical..

